# Peer Review of “Development and Assessment of a Point-of-Care Application (Genomic Medicine Guidance) for Heritable Thoracic Aortic Disease”

**DOI:** 10.2196/63645

**Published:** 2024-10-08

**Authors:** Anonymous

**Keywords:** genomic medicine, point of care, thoracic aortic aneurysm, aortic dissection, decision support

*This is a peer-review report for “Development and Assessment of a Point-of-Care Application (Genomic Medicine Guidance) for Heritable Thoracic Aortic Disease.”*

## Round 1 Review

### General Comments

The Genomic Medicine Guidance application described in this paper [1] needs to be made more intuitive for patients and clinicians to use. Additionally, genetic data integration needs to be expanded, clinical recommendations based on updated thoracic aortic aneurysms and dissections guidelines need to be updated frequently, and patient education materials need to be improved for clarity. It is also essential to make improvements for user feedback methods, multilingual support, accessibility, and strong data security. Enhancing the app’s effectiveness and usability requires a number of improvements, including customizable report options, better electronic health record integration, mobile device optimization, extensive training materials for clinicians, new research alerts, interactive tools like risk calculators, enhanced app performance, collaborative features, scalability to handle increased data loads, telehealth integration, support for custom user profiles, and community support features.

### Specific Comments

#### Major Comments

1. How can the user interface be improved so that patients and physicians find it easier to use?
2. How can the application include a wider variety of genetic data sources?
3. How are clinical recommendations updated on a regular basis in accordance with the latest thoracic aortic aneurysms and dissections guidelines?
4. What improvements can be made to the application’s patient education resources to improve understanding?
5. What changes are required to the application to increase its accessibility for users with disabilities?
6. Is multilingual assistance for people who don’t speak English planned?
7. How successful is the existing system for collecting user input, and how may it be improved?
8. What more privacy and data security precautions are required?
9. Does the application offer extra choices for customizing report generation?
10. What improvements may be made to the application’s integration with other electronic health record systems?
11. How can the performance of the application be enhanced on mobile devices?
12. Are there enough resources available to teach practitioners who are not experienced with interpreting genetic data?
13. Could the application be coupled with an alert system for fresh study findings?
14. What interactive features (risk calculators, for example) can be included in the application?
15. How might the application’s general functionality and speed be enhanced?
16. What further elements may be included to promote cooperation between medical professionals and patients?
17. As the application grows, how will it handle higher user and data loads?
18. Is it feasible to incorporate telehealth functionalities for conducting distant consultations?
19. Is it possible for the application to accommodate unique user profiles for various user groups, such as patients, researchers, and doctors?
20. How can the application encourage the exchange of information and experiences through a community support feature?
